# Resilience and Posttraumatic Growth of Patients With Breast Cancer During the COVID-19 Pandemic in China: The Mediating Effect of Recovery

**DOI:** 10.3389/fpsyg.2021.811078

**Published:** 2022-01-20

**Authors:** Jing Shi, Kristin K. Sznajder, Shuo Liu, Xinyue Xie, Xiaoshi Yang, Zhen Zheng

**Affiliations:** ^1^Department of Medical Oncology, The First Affiliated Hospital of China Medical University, Shenyang, China; ^2^Department of Public Health, College of Medicine, Pennsylvania State University, Hershey, PA, United States; ^3^Department of Social Medicine, College of Health Management, China Medical University, Shenyang, China; ^4^Department of Intensive Care Unit, Cancer Hospital of China Medical University, Liaoning Cancer Hospital and Institute, Shenyang, China

**Keywords:** posttraumatic growth, resilience, recovery, breast cancer, COVID-19

## Abstract

**Purpose:**

This study aims to examine the mediating role recovery plays in the relationship between resilience and posttraumatic growth (PTG) among breast cancer patients.

**Methods:**

A cross-sectional study design was implemented between January 02, 2021 and April 29, 2021. A total of 789 breast cancer patients from eight hospitals in Liaoning province were selected for participation in this study. These participants completed questionnaires, which included the Post-Traumatic Growth Inventory, EGO Resilience Scale and the Questionnaire about the Process of Recovery. The associated factors of PTG were analyzed using hierarchical multiple regression (HMR). The proposed relationships among resilience, recovery, and PTG were checked by structural equation modeling (SEM) analyses.

**Results:**

The average PTG score of breast cancer patients was 53.00 ± 28.30. PTG was positively correlated with both recovery and PTG (a*b = 0.1, BCa95% CI: 0.154 ∼ 0.054).

**Conclusion:**

Breast cancer patients were found to exhibit a moderate degree of PTG. Resilience was positively associated with PTG and recovery mediated the positive effect of resilience on PTG. Resilience might serve as a crucial protective factor that could explain positive growth in life-threatening illnesses through the mediating path of recovery.

## Introduction

Among women, breast cancer is one of the top five prevalent cancers worldwide and the mortality rate rises every year. According to the World Health Organization, the incidence and mortality of breast cancer are increasing and nearly 1 in 10 women have suffered from breast cancer each year around the world (Liu et al., 2018). Since the end of the 20th century, an increasing trend of breast cancer incidence has been observed among Chinese women, and the global incidence has more than doubled (Fan et al., 2014; Liu et al., 2018). Breast cancer incidence among Chinese women is the highest in the world (Jemal et al., 2007; Fan et al., 2014), which is underlined by a recent study that showed 420,000 Chinese women were diagnosed with breast cancer in 2020, account for 20% of all new cancer cases in China (Jemal et al., 2007; Fan et al., 2014).

As a stressful life event, the breast cancer diagnosis not only has a great impact on physical health but also has a detrimental influence on psychological health. Negative psychological health conditions like depression and anxiety are linked with elevated symptoms of cancer burden, decreased quality of life, and adverse health outcomes (Fann et al., 2008). Breast cancer, as a life-threatening life event, may cause distress and decreased quality of life. However, recent studies have shown that in addition to distress, vital life event or trauma could also bring about positive effects like post-traumatic growth (PTG) on patients (Zhai et al., 2019). In the process of adapting to trauma or vital stressful life event, PTG has been found to elicit positive psychological changes and personal growth. PTG is a positive psychological struggle with trauma and adaption that an individual develops after experiencing a traumatic life event (Scrignaro et al., 2016). It is also a subjective perception of positive change, which is reflected in several aspects such as mental state, life satisfaction, and interpersonal relationships (Cormio et al., 2015) and is conducive to the individual’s health. Patients who suffer from cancer experience a major life-threatening event, which may catalyze PTG to some degree. Further, prior literature demonstrates that individuals with higher PTG tend to report lower levels of depressive symptoms (Soo and Sherman, 2015). At the same time, PTG has been found to be related to lower pain, higher quality of life, greater overall well-being, and a longer life expectancy among cancer patients (Hill and Watkins, 2017). Most studies focus on the negative experiences of cancer survivors; however, PTG, is critically important to understand the adaption process whereby individuals exhibit positive self-growth related to trauma, as noted in positive psychology theory (Stockton et al., 2011). Therefore, elucidating PTG in patients with breast cancer is critical to improve the lives for cancer survivors. PTG is regarded as a “positive psychological change experienced as a result of the struggle with highly challenging life circumstances” in accordance with the model of Tedeschi and Calhoun (2004). PTG is thought to elucidate positive psychological changes and personal growth in oneself due to experiencing a trauma, and PTG may be increased by individual positive psychological resources and cognitive processing (Gori et al., 2021a). The prior literature also demonstrates that women with breast cancer may combine cognitive strategies to adjust their actions and accept the cancer diagnosis in a positive way (Carver, 1998; Molina et al., 2014; Romeo et al., 2019).

Positive factors such as resilience can increase PTG by taking advantage of positive psychological resources. Resilience is considered to be a coping strategy in the face of life adversities and refers to the capability of maintaining stable psychological functioning when exposed to a stressful or traumatic event especially one that lasts for a long time, such as breast cancer (Dalum et al., 2015; Li et al., 2019). High resilience, which indicates that the individual has the capability to positively regulate his or her physical and psychological health in the face of challenges, can bring about more positive resources to enhance PTG (Ding et al., 2020). After being diagnosed with breast cancer, patients inevitably experience distress or traumatic stress, and resilience can help them “bounce back” from stressful experiences quickly to alleviate symptoms of distress and enhance PTG by increasing positive psychological resources (O’Dowd et al., 2018). Prior literature also notes that positive resources could help individuals persist and deal with traumatic difficulties through the resiliency process, and thus enhance PTG. Previous studies have found a positive association between resilience and PTG for cancer survivors (Davydov et al., 2010), while, some literature indicated that there was no association between PTG and resilience.

Recovery is an underlying positive psychological resource which can help people return to pre-stressor status and is thought to bring about positive resources. Furthermore, recovery is recognized as a national mental health intervention and widely applied in most western countries (Tedeschi and Calhoun, 1996). The process of recovery offers a transformative conceptual framework for psychological health interventions in clinical practice and health care service delivery (Tian and Hong, 2013). With higher recovery, patients have more positive resources and positive emotions which can help breast cancer patients face trauma and enhance PTG. Studies have found that the greater resilience of cancer patients, the greater their recovery (Sonnentag and Fritz, 2007). Further, recent research depicts that recovery can be enhanced by providing recovery training to cancer patients [12]. Prior research shows that recovery plays a mediating effect between burnout and life satisfaction (Song et al., 2021). Patients with higher resilience may strive to reduce the loss of personal resources and gain positive outcomes via the process of recovery to enhance more favorable outcomes such as PTG, which is consistent with the recent literature indicating recovery mediates the relationship between resilience and HRQOL in physicians (Ding et al., 2020). For patients with breast cancer, resilience may also lead to improved mental health and treatment outcomes by reducing or mitigating the shock of cancer and the disadvantageous impacts of adverse life events (Tian and Hong, 2013), which will promote the patients’ process of recovery and PTG. Thus, recovery is hypothesized to enhance the development of PTG and mediate the association between resilience and PTG among breast cancer patients.

Although there has been a dearth of research on the association between resilience, recovery and PTG, this study aims to estimate the levels of PTG in breast cancer patients and examine the mediating role of recovery on the positive effect of resilience on PTG. This study hypothesized that (1) resilience and recovery are positively associated with PTG and (2) recovery plays a mediating role between resilience and PTG among breast cancer patients in China.

## Materials and Methods

### Participants and Procedure

From January 02, 2021 to April 29, 2021, a cross-sectional study was implemented among patients with breast cancer in Liaoning province, China. Data were gathered using face-to-face interviews based on a questionnaire comprised of questions on demographic characteristics, Posttraumatic Growth Inventory (PTGI), 14-item EGO Resilience Scale (RS-14), and the Process of Recovery (QPR). About 850 patients with breast cancer from eight hospitals in Liaoning province were recruited for this study according to the inclusion and exclusion criteria as follows: the inclusion criteria covered: (1) aged 18 years old or older; (2) diagnosed with breast cancer by imageological examination in accordance with clinical diagnostic criteria; (3) voluntarily participated in the study. The exclusion criteria covered: (1) experiencing cognitive dysfunction, visual dysfunction or other impairments which restrained participants from completing the questionnaires independently (mental disorder was self-reported by the participants themselves or with the diagnosis by the specific doctors in the medical records of the hospitals, and cognitive dysfunction was based on the definite diagnosis by the doctors); (2) diagnosed with mental disorders and undergoing medical therapies. A total of 789 patients took part in this study, contributing to a valid response rate of 92.82%.

### Ethics Statement

This survey was implemented in accordance with the Helsinki Declaration (1989). The Ethics Committee at China Medical University approved this study (CMU1210400061). The aims and contents of this study were explained to each participant and written informed consent was obtained from the breast cancer patients before beginning the survey.

### Measures

#### Demographic Characteristics

The measurement of patient demographic characteristics included age (<55 years, ≥55 years), marital status (married, other), education (high school and below, some college and above), monthly income [<3,000 ($463 USD) yuan, ≥3,000 yuan], medical insurance (urban worker, other), household location (urban area and rural area), chronic disease history, and the impact of COVID-19. Chronic disease was categorized as “yes” if they had ever been diagnosed with a common chronic disease (e.g., hypertension, cardiovascular disease, diabetes, and arthritis).

The impact of COVID-19 was assessed by “Impact on the approach of treatment due to COVID-19” (yes, no), “Delay or interruption of treatment due to COVID-19,” “Delay of hospitalization due to COVID-19,” “Impact on finances due to COVID-19,” “Impact on daily life due to COVID-19,” and “Impact on care from family due to COVID-19.”

#### Measurement of Posttraumatic Growth

The Posttraumatic Growth Inventory (PTGI), consisting of 21 items, was employed to measure PTG (Romeo et al., 2017) and was widely used in the Chinese population (Dong et al., 2017). Responses to questions on the PTGI are given through a six-point Likert scale with 0 point (I did not experience this change) to 5 points (I experienced this change to a very great degree). An overall score of PTGI was calculated as the summation of all points for each question. The higher scores implied higher levels of PTG. This scale was found to have a high Cronbach’s alpha coefficient of PTGI (0.974), which indicated good reliability.

#### Measurement of Resilience

Resilience was estimated by the 14-item EGO Resilience Scale (RS-14), which is commonly used to measure levels of resilience (Kim et al., 2020). Each item was assessed by a seven-point Likert scale with 1 point (strongly disagree) to 7 points (strongly agree). Each item was summed for an overall score, with higher scores indicating better resilience. This scale has been widely used in Chinese population (Ma et al., 2021). This scale had a high Cronbach’s alpha coefficient (0.928), which indicated good reliability.

#### Measurement of Recovery

Recovery was measured by the Questionnaire about the Process of Recovery (QPR) (Sonnentag and Fritz, 2007). The scale consists of 16 items with a 5-point Likert scale from 1 point (do not agree at all) to 5 points (fully agree). Higher scores indicate greater recovery. This scale had a high Cronbach’s alpha coefficient (0.970), which indicated good reliability. The QPR scale has been widely used in Chinese population in my previous study (Yang et al., 2020).

### Statistical Analysis

Statistical Package for the Social Science (SPSS) version 23.0 (IBM Corporation) and AMOS 17.0 were employed to perform the statistical analysis. *T*-tests and one-way analysis of variance (ANOVA) were implemented to depict the differences in PTG among categorical variables. Pearson correlation coefficients were tested to measure the correlation of PTG, resilience, and recovery. Three blocks of independent variables with the incremental variance of each block were presented to elucidate the associated factors of PTG with hierarchical multiple regression (HMR) analyses. PTGI scores served as the continuous dependent variable with the independent variables modeled in three procedures: Procedure 1: Demographic characteristics of the patients with breast cancer; Procedure 2: Resilience; Procedure 3: Recovery. Standardized parameter estimates (standardized β) were assessed to examine the magnitude of associations across the independent variables. The fitting of the model was evaluated by the *R*^2^–value.

Structural equation modeling (SEM) was employed to depict the mediating function of recovery on the positive effect of resilience on PTG. PTG, resilience, and recovery were entered as the dependent variable, independent variable, and the mediator, respectively, with the bias-corrected and accelerated 95% CI (BCa 95% CI) for each a*b product tested in SEM model. The model should be met with the indicators as followings: χ^2^/df less than 5, GFI higher than 0.90, CFI higher than 0.90, TLI higher than 0.90, and RMSEA less than 0.08. The bootstrapping was carried out to elucidate the mediating effect of recovery. A two-tailed probability value less than 0.05 implied statistically significant findings.

## Results

### Demographic Characteristics

Table 1 presented the demographic characteristics and COVID-19 related information of patients with breast cancer. All of the participants were women and aged 53.28 ± 10.82 on average. More than half the participants reported that the COVID-19 pandemic impacted on their finances (67.55%, 533/789), daily life (75.41%, 595/789), and care from family (67.05%, 529/789). It was observed that participants who reported impact on daily life due to COVID-19 had significantly higher scores of PTG (*P* = 0.003).

**TABLE 1:** Demographic characteristics of patients with breast cancer and PTG distribution.

Variables	*N*(%)	Resilience Mean ± SD	Recovery Mean ± SD	PTG Mean ± SD
**Age (year)**				
< 55	421(53.36)	39.98 ± 9.71**	68.61 ± 13.00**	57.85 ± 26.70**
≥ 55	368(46.64)	36.55 ± 10.89	63.19 ± 14.71	47.45 ± 28.47
**Marital status**				
Married	721(91.38)	38.29 ± 10.30	65.99 ± 14.18	53.05 ± 28.11
Other	68(8.62)	39.33 ± 11.54	67.11 ± 13.02	52.46 ± 27.08
**Education**				
High school and below	355(44.99)	37.84 ± 11.25	65.55 ± 14.67	50.95 ± 29.73
College and above	434(55.01)	38.82 ± 9.67	66.52 ± 13.58	54.67 ± 26.43
**Monthly income (¥)**				
<3,000	385(48.80)	37.56 ± 11.52**	64.97 ± 15.49*	50.28 ± 29.51*
≥3,000	404(51.20)	39.16 ± 9.19	67.15 ± 12.52	55.59 ± 26.27
**Medical insurance**				
Urban worker	465(58.94)	38.36 ± 10.21	66.27 ± 14.21	53.15 ± 28.06
Other	324(41.06)	38.40 ± 10.72	65.81 ± 13.91	52.78 ± 27.97
**Location**				
Urban area	490(62.1)	38.37 ± 10.24	66.27 ± 14.23	53.19 ± 28.05
Rural area	299(37.9)	38.39 ± 10.70	65.77 ± 13.84	52.70 ± 27.99
**Chronic disease**				
Yes	461(58.43)	37.91 ± 10.01	65.28 ± 13.32	52.70 ± 27.05
No	328(41.57)	39.04 ± 10.93	67.21 ± 15.03	53.42 ± 29.33
**Impact on approach of treatment due to COVID-19.**				
Yes	383(48.54)	38.03 ± 9.41	65.83 ± 11.80	53.71 ± 27.28
No	406(51.46)	38.71 ± 11.28	66.33 ± 15.95	52.33 ± 28.69
**Delay or interruption of treatment due to COVID-19.**				
Yes	219(27.76)	38.21 ± 9.43	64.94 ± 11.84	55.19 ± 26.54
No	570(72.24)	38.44 ± 10.76	66.52 ± 14.84	52.16 ± 28.53
**Delay of hospitalization due to COVID-19.**				
Yes	221(28.01)	38.27 ± 9.73	64.70 ± 11.96	54.57 ± 26.89
No	568(71.99)	38.42 ± 10.67	66.62 ± 14.80	52.39 ± 28.43
**Impact on finance due to COVID-19.**				
Yes	533(67.55)	38.10 ± 9.93	66.37 ± 12.79	54.06 ± 26.99
No	256(32.45)	38.96 ± 11.34	65.49 ± 16.45	50.79 ± 29.95
**Impact on daily life due to COVID-19.**				
Yes	595(75.41)	38.26 ± 9.80	66.19 ± 13.42	54.71 ± 26.52**
No	194(24.59)	38.75 ± 12.13	65.76 ± 15.97	47.77 ± 31.64
**Impact on care from family due to COVID-19.**				
Yes	529(67.05)	37.80 ± 9.85*	64.86 ± 14.16**	53.72 ± 26.56
No	260(32.95)	39.56 ± 11.41	68.56 ± 13.62	51.53 ± 30.74

*Significant at the 0.05 level (two-tailed); **significant at the 0.01 level (two-tailed).

### Correlations Among Posttraumatic Growth, Resilience and Recovery

The results of the Pearson correlation analysis of PTG, age, resilience, and recovery are listed in Table 2. PTG was positively correlated with both resilience and recovery (*P* < 0.01). Additionally, resilience had a positive correlation with recovery (*P* < 0.01).

**TABLE 2:** Correlations of PTG, age, resilience, and recovery.

	*M*	*SD*	1	2	3	4
(1) PGT	53.00	28.00	1			
(2) Age	53.28	10.60	−0.148**	1		
(3) Resilience	38.38	10.41	0.307**	−0.064	1	
(4) Recovery	66.08	14.08	0.366**	−0.155**	0.500**	1

**Significant at the 0.01 level (two-tailed).

### Regression Analysis of Posttraumatic Growth, Resilience, and Recovery

**FIGURE 1:**
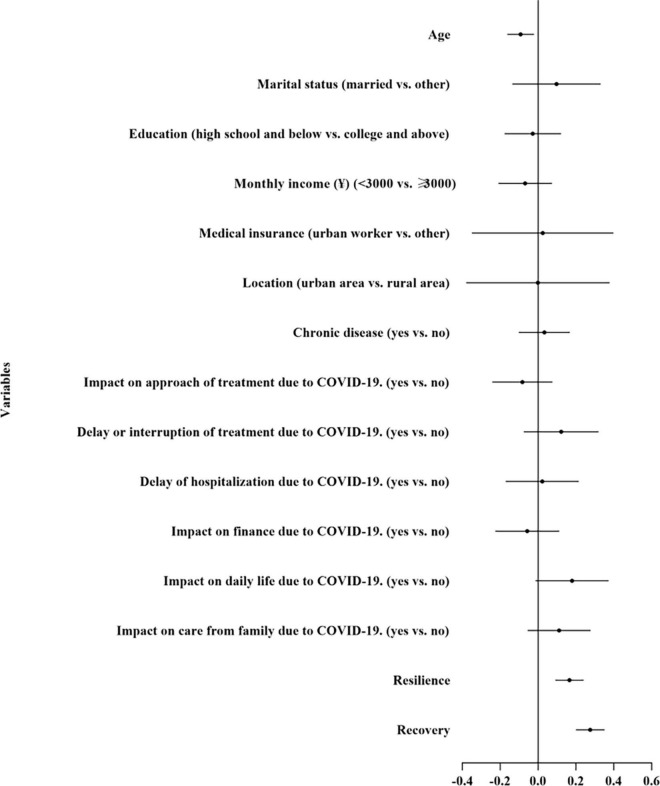
Forest plot of associated factors of posttraumatic growth.

Table 3 and Figure 1 illustrate the results from the HMR. A total of 18.0% of the variance was explained by the final HMR model, with 8.8 and 5.5% of the variance explained by resilience and recovery, respectively. Both resilience (β = 0.166, 95% CI = 0.091–0.240, *P* < 0.01) and recovery (β = 0.276, 95% CI = 0.200–0.351, *P* < 0.01) were observed to increase the odds of PTG.

**TABLE 3:** Associated factors of PTG during the COVID-19 pandemic.

Variables	Model 1			Model 2			Model 3		
	β	Standardized β	95%CI	β	Standardized β	95%CI	β	Standardized β	95%CI
**Block 1: Demographic characteristics of patients with breast cancer**									
Age	−0.133**	−0.133**	−0.208 – −0.057	−0.121**	−0.121**	−0.193 – −0.048	−0.092*	−0.092*	−0.163 – −0.022
Marital status (married vs. other)	0.062	0.017	−0.191–0.315	0.098	0.027	−0.143–0.339	0.098	0.027	−0.136–0.331
Education (high school and below vs. college and above)	−0.018	−0.009	−0.179–0.143	−0.005	−0.003	−0.159–0.149	−0.028	−0.014	−0.177–0.121
Monthly income (¥) (<3,000 vs. ≥3,000)	−0.14	−0.07	−0.292–0.013	−0.091	−0.045	−0.237–0.055	−0.068	−0.034	−0.210–0.073
Medical insurance (urban worker vs. other)	0.038	0.018	−0.368–0.443	0.044	0.022	−0.342–0.431	0.024	0.012	−0.350–0.399
Location (urban area vs. rural area)	−0.018	−0.009	−0.428–0.392	−0.003	−0.001	−0.394–0.388	−0.001	−0.001	−0.380–0.378
Chronic disease (yes vs. no)	−0.008	−0.004	−0.154–0.138	0.023	0.011	−0.117–0.162	0.033	0.016	−0.102–0.168
Impact on approach of treatment due to COVID-19. (yes vs. no)	−0.078	−0.039	−0.250–0.093	−0.060	−0.030	−0.224–0.104	−0.083	−0.042	−0.242–0.076
Delay or interruption of treatment due to COVID-19. (yes vs. no)	0.115	0.052	−0.099–0.329	0.109	0.049	−0.095–0.313	0.122	0.055	−0.075–0.320
Delay of hospitalization due to COVID-19. (yes vs. no)	0.012	0.006	−0.196–0.221	−0.007	−0.003	−0.206–0.192	0.022	0.010	−0.171–0.215
Impact on finance due to COVID-19. (yes vs. no)	−0.039	−0.018	−0.221–0.144	−0.018	−0.008	−0.192–0.156	−0.057	−0.027	−0.226–0.112
Impact on daily life due to COVID-19. (yes vs. no)	0.225*	0.097*	0.016–0.435	0.206*	0.089*	0.006–0.406	0.179	0.077	−0.014–0.373
Impact on care from family due to COVID-19. (yes vs. no)	−0.014	−0.007	−0.193–0.165	0.039	0.019	−0.132–0.211	0.111	0.052	−0.056–0.278
**Block 2. Resilience**				0.299**	0.299**	0.233–0.366	0.166**	0.166**	0.091–0.240
**Block 3. Recovery**							0.276**	0.276**	0.200–0.351
***R* ^2^**		0.038			0.126			0.180	
**Adjusted *R*^2^**		0.022			0.110			0.164	
**Δ*R*^2^**		0.038			0.088			0.055	

*Significant at the 0.05 level (two-tailed); **significant at the 0.01 level (two-tailed).

### Recovery as a Mediator Between Resilience and Posttraumatic Growth

**FIGURE 2:**
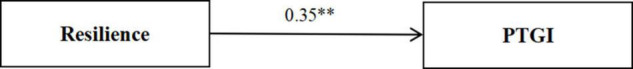
Standardized solution for the structural equation model of resilience and posttraumatic growth. **Significant at the 0.01 level (two-tailed).

The variables included in the SEM were based on the HMR results (Figure 2). The total effect of resilience on PTG was measured first (*c* = 0.35) by a model with good model fit indices (χ^2^/df = 2.671 < 5, GFI = 0.953 > 0.90, AGFI = 0.931 > 0.90, CFI = 0.981 > 0.90, TLI = 0.975 > 0.90, RMSEA = 0.046 < 0.05).

**FIGURE 3:**
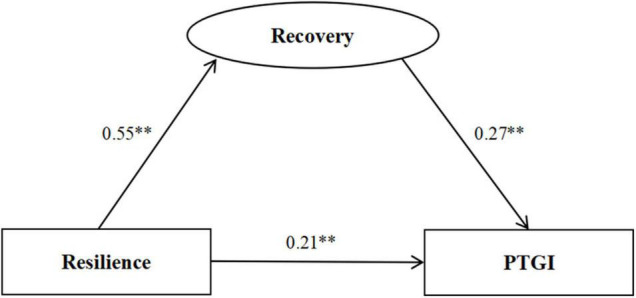
Standardized solution for the structural equation model of resilience, recovery, and posttraumatic growth. **Significant at the 0.01 level (two-tailed).

Figure 3 presents the indirect effect of resilience on PTG via recovery (a*b = 0.14) with a model of good model fit indices (χ^2^/df = 2.397 < 5, GFI = 0.949 > 0.90, AGFI = 0.930 > 0.90, CFI = 0.982 > 0.90, TLI = 0.978 > 0.90, RMSEA = 0.042 < 0.05). The effect of resilience on PTG was still significant (*c*′ = 0.21, *P* < 0.001) when recovery was treated as a mediator (BCa 95% CI = 1.160–2.596, 95% CI = 1.160–2.596), which indicating that the mediating function of recovery was significant in the relationship between resilience and PTG.

## Discussion

### Principal Findings

To date, this is the first study to elucidate the mediating function of recovery between resilience and PTG among breast cancer patients. The present study found that the PTG score among patients with breast cancer in China during the COVID-19 pandemic was at a moderate level (53.00 ± 28.30). Further, PTG scores among patients with breast cancer in this study were similar to those in another study of patients with breast cancer (53.8 ± 21.90) before the COVID-19 pandemic (Romeo et al., 2017), but lower than those of patients with glioma (63.36 ± 21.44) (Kim et al., 2020) and Chinese women diagnosed with gynecological cancer (56.5) (Zhou et al., 2021). From the results, it can be clearly seen that breast cancer patients may not only experience negative mental health outcomes, but also may experience benefits such as the development of PTG during the adjustment to a cancer diagnosis. Patients with breast cancer possibly confront hardships and suffering during the diagnosis and treatment of their illness. However, they may also experience positive changes and psychosocial growth, such as the development of PTG including an increased appreciation of life, greater sense of personal strength and self-understanding, renewed appreciation for intimate relationships, and positive spiritual changes. Patients with breast cancer who experience PTG might experience improved mental toughness, from the adjusting to their cancer diagnosis, increased adaption, and improved clinical outcomes.

The impact on daily life due to COVID-19 was a positive predictor of PTG as shown in the results of HMR Models 1 and 2, which indicated the PTG of the patients increased when COVID-19 influenced the patients’ daily life and cancer treatment. Patients who reported that COVID-19 impacted on their daily life were found to have higher levels of PTG and higher levels of recovery. This is likely because those patients gained more PTG through the process of dealing with the COVID-19 pandemic related stress and the positive impact of recovery.

Cancer patients need to visit hospitals frequently for various examinations and chemotherapy, which greatly elevates their risk of COVID-19, and may result in the development of PTG (Al-Shamsi et al., 2020). A study showed that cancer patients had twice the odds of contracting SARS-CoV-2 compared to the public (Yu et al., 2020). Thus, the increased risk of SARS-CoV-2 infection and a pre-existing cancer diagnosis may have accelerated PTG. The patients who reported that COVID-19 impacted on their daily life could have made positive and meaningful psychological changes as a result of struggling with breast cancer and may have exhibited a more positive attitude to daily life so that they might have a calm and rational view on the COVID-19 pandemic and thus triggering PTG.

In this study, we observed from the final HMR Model 3, resilience and recovery were both positively associated with PTG, with recovery acting as the mediator between resilience and PTG among breast cancer patients. Resilience was a positive predictor of PTG which was consistent with previous research, but inconsistent with several other studies showing no correlations between PTG and resiliency which might be due to the various population estimates and different psychological processes of patients in a variety of settings (Walsh et al., 2018). Resilience is a key component of positive psychological resources, which could improve cognitive processing and positive coping when an individual is confronted with a trauma. According to the thriving model of illness by Carver, resilience can decrease the impact of subsequent stressors on patients and PTG can be accompanied with resilience and help patients return to a healthy functional level (Carver, 1998). Resilient breast cancer survivors were observed to have greater PTG in our study, which is in accordance with previous research (Dong et al., 2017). Past research has shown that resilience can be elevated by enhancing family and social support, which can improve an individual’s problem-solving ability and resilience can also be elevated by participating in physical activity (Li et al., 2019).

In addition, resilience seems to play an important functioning role in the development of PTG, as reported in prior study, which shows that the higher the patient’s resilience is, the easier it is to perceive PTG. A large number of studies revealed that high resilience could facilitate the patients’ adaptation to traumatic life events by enhancing positive reappraisal and taking advantage of coping resources for stress (Hulbert-Williams et al., 2018; Gori et al., 2021b). Further, resilient patients might consider the trauma of their disease as an opportunity for personal growth and development and thus, they experience more PTG. Conversely, patients with less resilience, may experience a lower likelihood for PTG, as they may be trapped in distress and fear of disease.

In this study, we found that breast cancer patients with adequate recovery were more likely to experience high levels of PTG, which suggested that recovery could promote the development of PTG. Typically, PTG is thought to occur after patients’ experience of a traumatic, stressful, or significant life changing event (Paredes and Pereira, 2018). Yet, other studies suggest that trauma recovery may be an important prerequisite. According to recent research, the more successful patients’ recovery, the higher the levels of mental growth and the greater the ability to identify and consider new life possibilities among patients with drug addition (Haroosh and Freedman, 2017). In addition, recovery also helps to regain hope, set new goals, and gain the courage to continue life for patients with severe mental illness (Ochocka et al., 2005). Similarly, breast cancer patients with optimal recovery may also tend to give positive evaluations about the diagnosis and treatment they have received, and actively cooperate with subsequent treatments and regain hopefulness, consequently promoting PTG.

Moreover, according to the results of the SEM, recovery has a mediating effect on the relationship between resilience and PTG, and higher levels of resilience increase the capability of recovery and enhance PTG through the mediating path of recovery. Resilience could facilitate quick recovery and positive adaptation of psychological health and maintain optimal health status after patients’ breast cancer diagnosis, and as a result, they would perceive the increased PTG (Li et al., 2019). A previous prospective study in burn patients found that PTG increased with the improvement of their recovery resources, as well as their health scores (Martin et al., 2017). Thus, feasible targets for prevention and intervention strategies should be provided to promote recovery in order to reduce the potential detrimental effects of trauma (Nitzan-Assayag et al., 2015). The process of recovery would promote the health conditions for patients to return to their previous states, and patients with adequate resilience who are adept at using their recovery experiences to improve their attitude, values, and goals, may be more likely to develop PTG. Through this process, people can live a more satisfactory life even when suffering from adversity according to the recovery model (Nitzan-Assayag et al., 2015).

Resilience is known as the active process of recovery and the capability to positively process threats, which could lead to the promotion of PTG (Tsai et al., 2015). The results were also in accordance with the theory of positive psychological resources, which indicate that resilience and recovery can increase positive coping and bring about favorable outcomes such as PTG. Therefore, attention should be paid to the process of recovery among breast cancer patients and resilience training should be conducted.

In this study, resilience and recovery were found to be positively associated with PTG and recovery mediated the positive effect between resilience and PTG. Recovery might serve as a crucial factor that explains positive growth among people who experience highly challenging life-threatening health problems. Enhancing cancer survivors’ resilience and recovery during treatment may facilitate their psychological adjustment and psychosocial functioning, thereby facilitating PTG stemming from their cancer diagnosis.

### Study Limitations

There are some limitations in this study. First, the study bears the limitation of its cross-sectional research design and therefore we cannot ascertain causality. Longitudinal research should be conducted in order to examine the positive effects of resilience and recovery on PTG. Second, other illness-related characteristics were not included in the study. Third, the use of self-reported measurements may result in social desirability bias. Finally, our study only included breast cancer patients in Liaoning province of China, which limits generalizability to all the cancer patients.

### Clinical Implications

In this study, breast cancer patients were found to have a moderate level of PTG during the COVID-19 pandemic. Resilience and recovery were positively associated with PTG and could enhance the development of PTG. Moreover, PTG could also be improved through the mediating path of recovery. The positive effects of resilience and recovery on cancer survivors should be fostered through positive psychological resources training in order to facilitate personal growth and positive changes for breast cancer patients.

## Data Availability Statement

The original contributions presented in the study are included in the article/supplementary material, further inquiries can be directed to the corresponding author.

## Ethics Statement

The studies involving human participants were reviewed and approved by the Ethics Committee at China Medical University. The patients/participants provided their written informed consent to participate in this study.

## Author Contributions

JS, KS, SL, XX, XY, and ZZ: study design. JS, SL, XX, and ZZ: data collection and acquisition. JS and XX: analysis of data. KS, SL, and XX: manuscript revision. All authors contributed to the design of this study.

## Conflict of Interest

The authors declare that the research was conducted in the absence of any commercial or financial relationships that could be construed as a potential conflict of interest.

## Publisher’s Note

All claims expressed in this article are solely those of the authors and do not necessarily represent those of their affiliated organizations, or those of the publisher, the editors and the reviewers. Any product that may be evaluated in this article, or claim that may be made by its manufacturer, is not guaranteed or endorsed by the publisher.
